# Some predictions of Rafael Lorente de Nó 80 years later

**DOI:** 10.3389/fnana.2014.00147

**Published:** 2014-12-03

**Authors:** Jorge A. Larriva-Sahd

**Affiliations:** Neuromorphology Lab, Department of Developmental Neurobiology, Instituto de Neurobiología, Universidad Nacional Autónoma de MéxicoQuerétaro, Mexico

**Keywords:** Lorente de Nó, cerebral cortex, summation coding, Ammon's horn, hippocampus

## Abstract

Rafael Lorente de Nó, the youngest of Santiago Ramón y Cajal disciples, was one of the last Century's more influential researches in neuroscience. This assay highlights two fundamental contributions of Rafael Lorente de Nó to neurobiology: the intrinsic organization of the mammalian cerebral cortex and the basic physiology of the neuron processes.

## Biographical note

**Figure 1:**
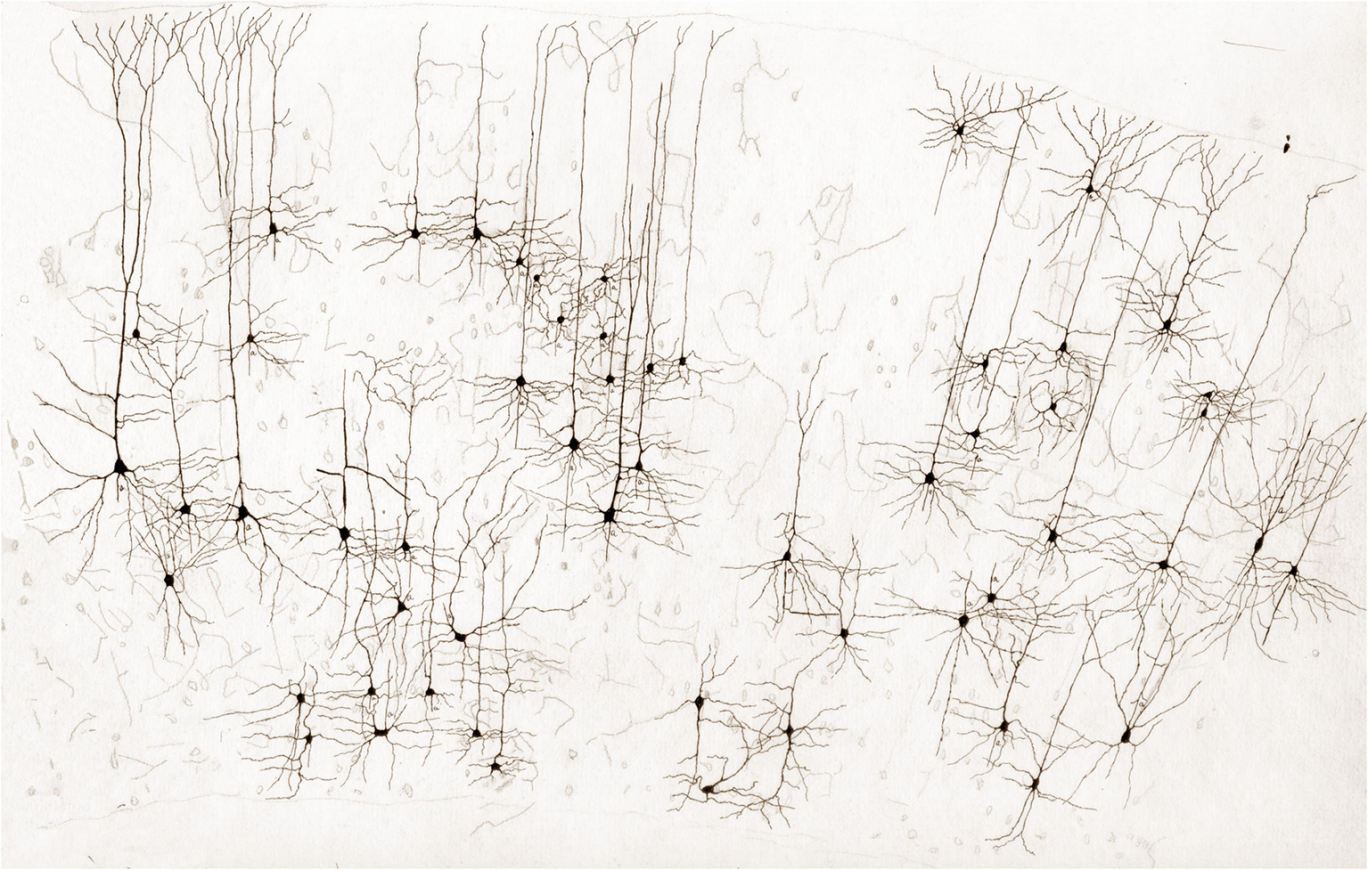
**Survey, unpublished drawing from the rat cerebral cortex by Lorente de Nó**. Golgi-Cox technique.

**Figure 2:**
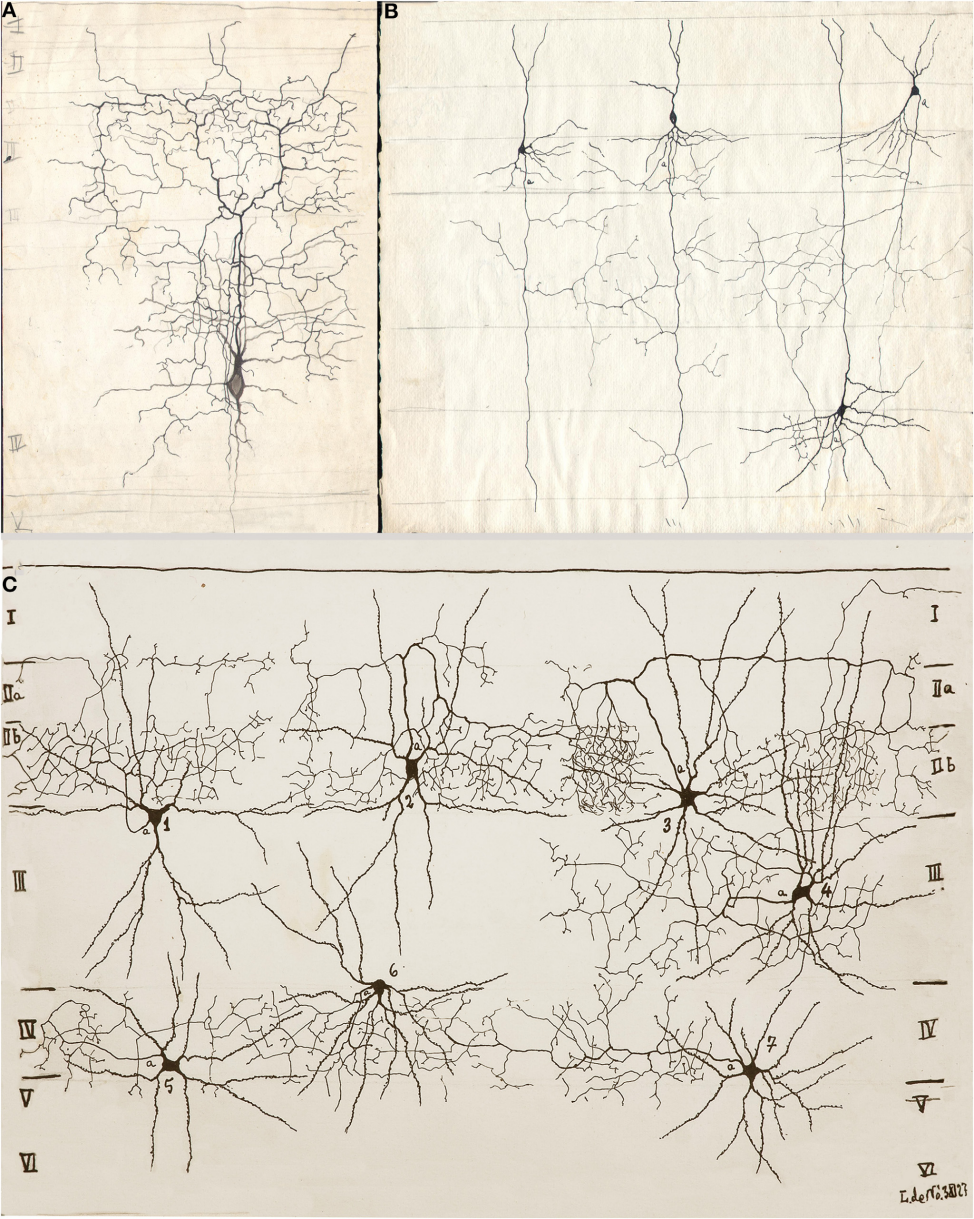
**Drawings from Lorente de Nó performed between 1922 and 1927 in the Ramón y Cajal laboratory. (A)** Short-axon neuron with an ascending axon that distributes primarily in layer III (III). **(B)** Three pyramidal cells with numerous axon collaterals to layer IV and an interneuron (upper right), whose descending axon (a) resolves in layer III. **(C)** Seven examples of shot-axon neurons (see Lorente de Nó, 1949). To note is that while the axon of each cell ramifies profusely, it remains confined to the homonymous layer. Newborn mice, rapid Golgi technique.

**Figure 3:**
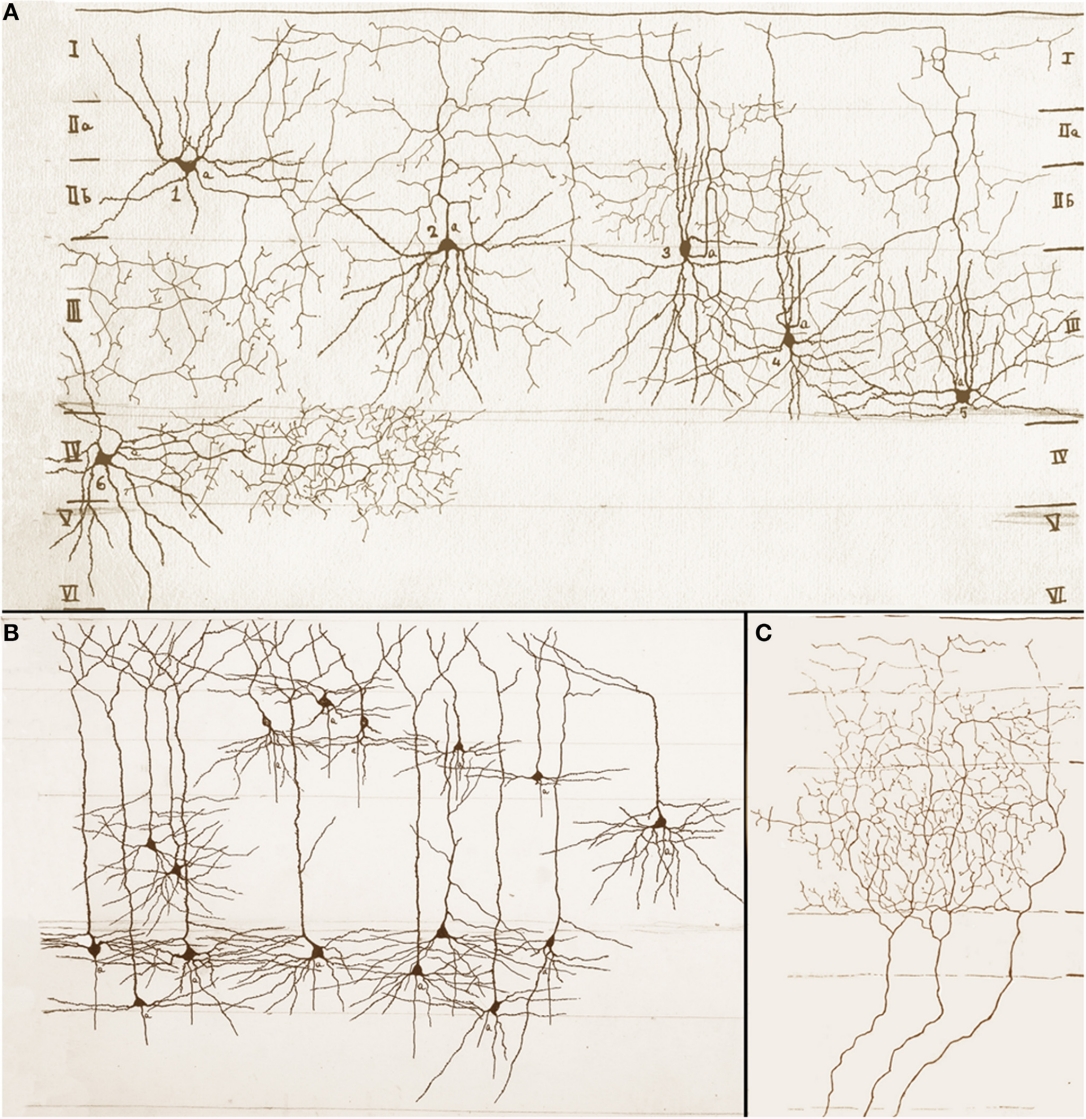
**Assorted drawings of the mouse cerebral cortex by Rafael Lorente de Nó. (A)** Examples of short-axon neurons that include ascending (cells 2, 4 and 5), descending (cell 1), and horizontal (cells 3 and 6) axons. Roman numbers at either side of the drawing designate cortical layers, which are bounded by soft pencil. **(B)** Examples of superficial and deep pyramidal cells; axons have been omitted. **(C)** Talamo-cortical fibers distributing throughout layers I to III (bounded with soft pencil).

Rafael Lorente de Nó stands out as one the most notable progeny that Santiago Ramón y Cajal bequeathed Neuroscience. A gifted, precocious intellectual, Lorente de Nó was born in Zaragoza, Spain, in the Kingdom of Aragón, on the 8th of April, 1902, and died in Tucson Arizona, on the 2nd of April, 1990. Initially guided by Pedro Ramón y Cajal, a Gynecologist and Professor of Histology at Zaragoza, Spain, Lorente de Nó learned from him the routine histological techniques and, notably, silver impregnations introduced earlier by Camilo Golgi himself. After this early exposure, Lorente de Nó traveled in 1920 to Madrid, where the bold 18-year-old is introduced to Santiago Ramón y Cajal. Following a memorable interview (Larriva-Sahd, 2002), Don Santiago, was perceptive enough to accept “paisano Lorente” in his laboratory as his youngest disciple. Soon afterwards Lorente de Nó embarks in his first studies on spinal cord regeneration (Lorente de Nó, 1921) and cerebral cortex (Lorente de Nó, 1922), obtaining the degree of Medical Doctor at the enviable age of 21. Among the most important studies Lorente de Nó carried out under the direct supervision of the Master are those relating to spinal cord regeneration, brain-stem nuclei and, especially, to the mouse cerebral isocortex, which resulted into 15 published works (see Larriva-Sahd, 2002). His experiments on the VIIIth cranial nerve earned him the attention of Nobel Laureate Robert Bárány, who was at that time in Spain visiting Cajal's lab (De Carlos and Pedraza, 2014). His experimental work on the vestibular system preceded his contribution to elucidating the structure of brainstem nuclei underlying cranial nerve reflexes. In 1924 Lorente de Nó traveled to Uppsala, and later to Berlin, where he worked with Robert Bárány, and with Oskar and Cecile Vogt, respectively. His most important studies on the brainstem nuclei and reflexes resulted from his interaction with Professor Bárány and were published in five different languages (i.e., French, Swedish, Russian, German, and English). In 1931 Lorente de Nó sailed to America and become head of the Neuroanatomical Laboratory at the Central Institute for the Deaf in St. Louis Missouri. In 1935 he was appointed lecturer at the Washington University, and the following year he moved to the Rockefeller Institute until his formal retirement in 1970. Lorente de Nó's most notable contributions to understand the physiology of nerve fibers and the microscopic organization of the cerebral cortex were performed during this period, although he later acknowledged that a substantial part of both ideas and results (Figures 1–**3**) of this work were conceived when he was still living in the Old Word (Larriva-Sahd, 2002). In the early 70's Lorente de Nó was invited by Dr. Victor Goodhill to join the University of California in Los Angeles, as a visiting Professor. From this late period resulted a long series of papers on the physiology of nerves and his detailed account on the VIII cranial nerve (Lorente de Nó, 1981). In 1981, he moved to Tucson Arizona where he died in 1990.

Lorente de Nó was member of the American Physiological Society and the American Association of Anatomists, the National Academy of Sciences, and the American Academy of Arts and Science. In addition to honorary degrees by the University of Uppsala, Clark University, and Rockefeller University, Lorente de Nó received the Karl Spencer Lushly Award, and the Award of Merit in 1986, and held an Honorary Membership at the UCLA-Brain Research Institute in 1972 (Woolsey, 2001).

It is debatable which of Lorente de Nó numerous contributions are the most relevant, even within the confines of a single publication; perhaps his most celebrated papers pertain to two fundamental areas of current neuroscience: his contributions to the modular organization of the mammalian cerebral cortex and to the basic physiology of the neurons. Since both topics were primarily presented by Lorente de Nó in his papers of the organization of the cerebral cortex, they are used here to provide a conceptual frame. Before his demise, Lorente de Nó left to this author a number of his original drawings; some dealing with different aspects of neocortical organization are presented here (Figures 1–**3**).

## Columnar organization of the mammalian cerebral cortex (Figures 1–4)

**Figure 4:**
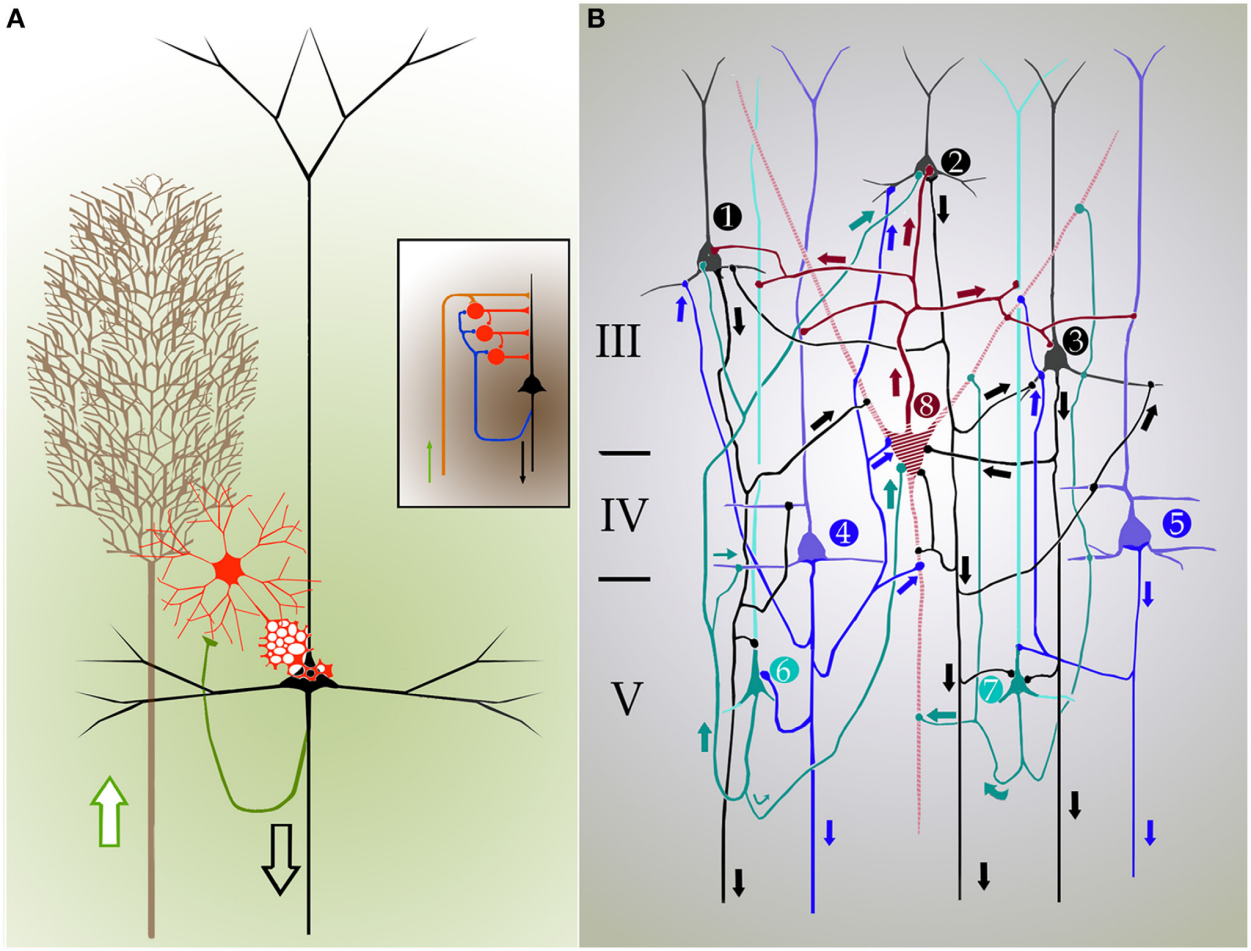
**Cartoons depicting the inner organization and synaptic interactions in the mammalian isocortex**. Arrows designate directionality of nerve impulses. **(A)** Cylindrical unit composed of afferent fibers (brown), short-axon (red), and pyramidal cells. Axon impulses arrive via talamo-cortical fibers (brown), and target short-axon neurons (red), and pyramids (black) that send efferent axons. A set of collaterals from the pyramidal cell axon (green) ascend back to the interneuronal pool and adjacent homologs. Inset. Synaptic interactions between individual components of the cortical elemental unit that represent the structural basis for cortical reflexes. **(B)** Diagram showing a closed, self-exciting chain in the entorhinalis cortex. 1, 2, 3–pyramids (black) of layer III; 4, 5–deep pyramids (blue); 6, 7–pyramids with recurrent axis cylinder of layer V (V). 8–cell with short axis cylinder of layer III (III). The discharge of pyramidal cells 1, 2, 3 and that of the small pyramids with recurrent axis cylinder (6,7) have raised in cell 8 a certain amount of central excitatory state (c.e.s.), and when the deep pyramids (4,5) discharge cell 8 reaches threshold c.e.s. The impulses of cell 8 excites again cells 1, 2, 3, that had already being facilitated by recurrent collaterals, and the cyclic process start again. The discharge of such closed chains will constitute the autogenous activity of the cortex; the frequency of impulses is dependent on the number of active links within the chain. **(A,B)** modified from Lorente de Nó (1933 and 1949).

Every researcher dealing with functional or developmental aspects of the mammalian cerebral cortex has a somewhat different idea on what a cortical column might be. In fact, rather than being a well-defined concept, the term cortical “column” is often a context-defined notion (Valverde, 1986; Mountcastle, 2003; Horton and Adams, 2005; Rockland, 2010; Merchant et al., 2012). While this issue is beyond the scope of the present brief assay, the term cortical “module,” or “elementary unit” was coined by Lorente de Nó to refer to a vertical neural quantum having distinct extrinsic afferences and interneurons converging onto single or a group of pyramidal cells (vide infra); the axon of the latter is both the output and recurring element of the unit. Clearly, at the time Lorente de Nó defined the existence of cortical modules (**Figure 4A** and insert), every element structuring them was previously identified (see Valverde, 1986). Thus, incoming fibers to the cerebral cortex were elegantly featured by Ramón y Cajal (1891) [also termed Ramón fibers by Kölliker (Ramón'sche Fasern)] (see Lorente de Nó, 1922) (**Figure 3C**); likewise, the existence of interneurons (Figures 2A,C, 3B) was defined earlier by C. Golgi and fully corroborated afterwards. The same applied to cortical pyramidal cells (see Jones, 1984) (Figures 1, 2B, 3B). Both cortical distribution and interaction between short-axon neurons and pyramidal cells were also noted by Ramón y Cajal himself. Furthermore, the notion of the central nervous system composed of linear series of neurons as understood by Sherrington and Ramón y Cajal prevailed until the 1930's (see Ramón y Cajal, 1904). While Lorente de Nó added an unprecedented number of novel cell-types and interactions among them, the basic ingredients that he utilized to develop his tenets on cortical organization were previously known. In a very straight forward way, Lorente de Nó conceived that geniality “is to utilize facts that have been previously neglected or assumed to be irrelevant for a given phenomenon” (Larriva-Sahd, 2002), and he did. Thus, he gathered (Figure 4A) previously characterized elements to advance two cardinal points, these are, their synaptic interactions–as reveled by the Golgi technique (see Wang et al., 2004)—and their potential function. Although it has been assumed (see Mountcastle, 2003) that the concept of cortical “module” was presented first in the memorable chapter released in 1949 (Lorente de Nó), this happened quite a while earlier. Time matters, especially because as earlier as 1933 he *anticipated* the existence of physiologically distinct elemental units nearly 20 years before that intracellular recordings came to our technological armamentarium. In fact, the essence of the concept created by Lorente de Nó imparts to structural elements a physiological implication: it is, therefore, binomial. Structurally, each vertical “elemental unit,” or “cylinder” is composed of afferent fibers, short-axon neurons and pyramidal cell(s) endeavor to produce a common physiological response. Indeed, what he wrote casts no doubt: “but no matter what kind of cylinders we choose, -all are equally well justified and everyone will have a real physiological existence at determined moments of cortical function” (Lorente de Nó, 1933). This furnished a tangible working hypothesis that, with the advent of electrophysiological studies, was successfully tested: recording electrodes coursing tangentially in the primary somatosensory (Mountcastle and Powell, 1959) and visual (Hubel and Wiesel, 1959) cortices proved that, the cerebral cortex is, in fact, composed of distinct vertical functional modules.

As previously stated, at the time Lorente de Nó performed his studies on the brainstem and cerebral cortex, the prevailing notion was that the central nervous system was composed of linear series of neurons. Fortunately, Lorente de Nó had to perform physiological studies in experimental models with small mammals paralleling his cytological studies, which led him to challenge this early concept. In this context, scrutiny of his work on the entorhinal cortex (Lorente de Nó, 1933) adds another fundamental observation, that is, the interaction between superficial and deep pyramidal cell layers (Figures 2A, 4B). While it had been recognized earlier that pyramidal cells in layer III (LIII) and deep layer V (LV) send projecting axons beyond the cortical confines, Lorente de Nó demonstrated that axon collaterals from pyramidal cells in either layer and those from pyramidal cells with ascending axons (Figure 4B, cells 6 and 7) converge on LIII interneurons and LIV pyramids themselves. This frequent axonal association and that from the short-axon neurons in LIII back to the adjacent pyramids (Figure 4B), led Lorente de Nó to suggest: “The activity of the cortex does not end with the first discharge of the pyramids, the afferent volley starts the autogenous activity of the cortex, that may be brought of [*sic*] in the following way (Figure 4B). The pyramids with recurrent axis cylinder (Figure 4B cells 6 and 7) and the deep pyramids (Figure 4B, cells 4 and 5) on the on hand, and the superficial pyramids (Figure 4B, cells 1, 2, and 3) on the other, constitute a closed chain of neurons, so that impulses may travel around, provided the refractory period of each cell is somewhat shorter than the time necessary for impulses to travel through the chain. For the chain superficial pyramids and pyramids with recurrent axis cylinder (alone), this condition can hardly be accomplished, but if one or several neurons with short axis cylinder (Figure 4B, cell 8) are intercalated the time relations within the chain will allow such autogenous activity.” As one might expect from Lorente de Nó postulation, voltage-sensitive optical visualization in slices from the cerebral cortex, two clusters of cellular activation would be detected following electrical stimulation of thalamic afferences. In fact, such stimuli (Laaris et al., 2000) revealed two foci that appear to correspond to supra- and infra-granular pyramids. Further, administration of the NMDA receptor antagonist to the medium attenuate significantly that response.

Regarding the concept of the central nervous system organized as longitudinal successions of neurons, Lorente de Nó (1933a) wrote what could be a closing statement: “The conception of the reflex arc as a unidirectional chain of neurons has neither anatomic nor functional basis. Histologic studies with Golgi's method show the universality of the existence of plural parallel connections and of recurrent, reciprocal connections. Study of vestibulo-ocular reflexes by isotonic recording of the eye muscles in the rabbit after various experimental lesions of the reflex centers leads to a physiologic interpretation in terms of closed “self-reexciting” chains of neurons. Nystagmus is an alternating reflex in which the peripheral labyrinthine stimulation sets into activity a machinery which gives rise to nystagmus in the same way that the spinal cord sets up the stretch reflex as a response to a skin stimulus. In the vestibular system there is no “localization” of reflexes in anatomic nuclei; the whole system is a functional unit; reflex reactions may be produced as long as the reflex arc is closed through one of the multiple pathways; on the other hand, lesions in any part of the vestibular system modify all the reflexes”(Lorente de Nó, 1933a). In précis, Lorente de Nó's identification of clusters of neurons linked by the sort of interaction just described, together with the inner circuitry of the tuberculum acousticum (Lorente de Nó, 1981) represent biological substrata to what latter on was known as a “feed-back loop.”

## Neuron as a time-space decoding device

**Figure 5:**
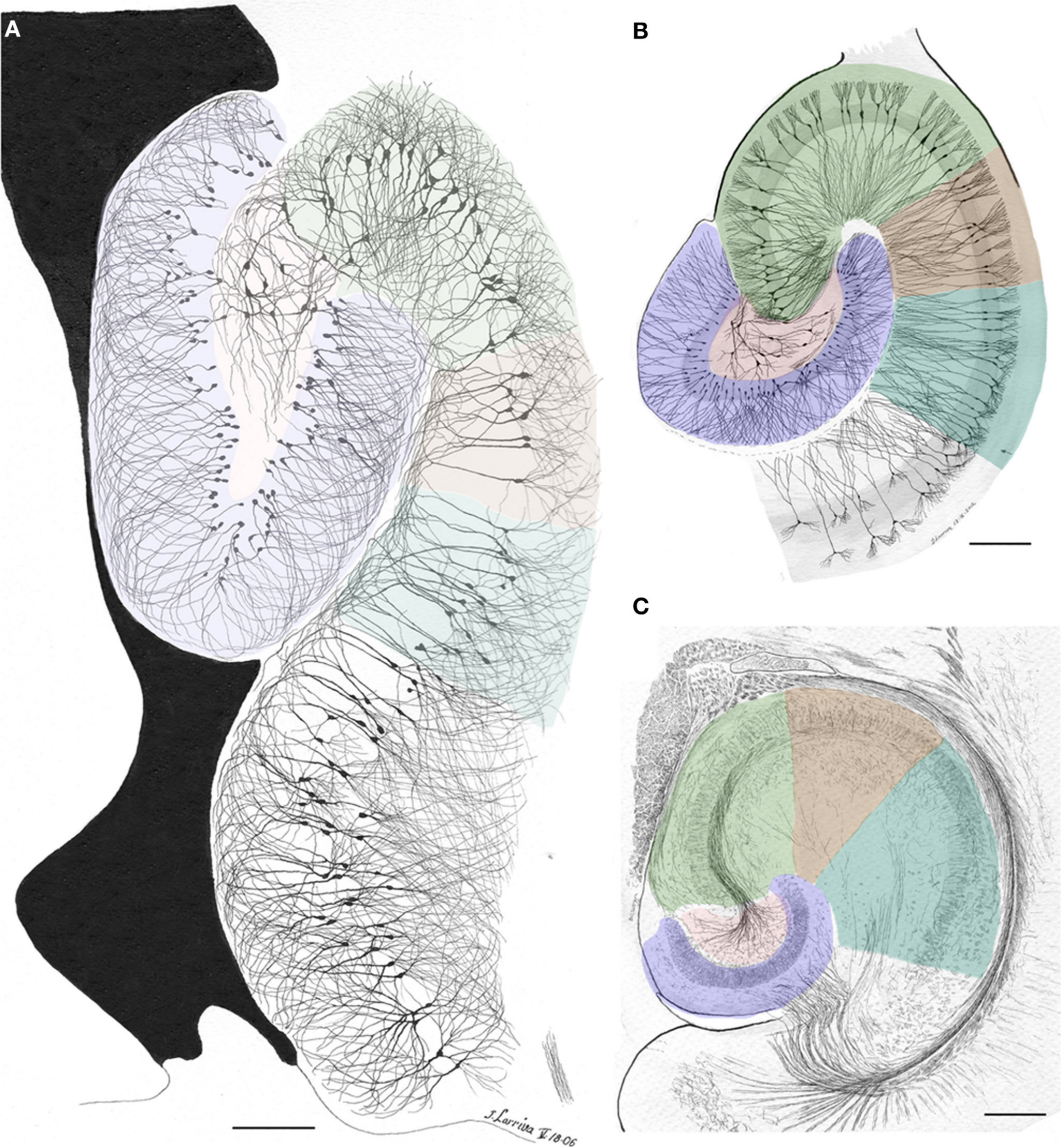
**Survey camera lucida drawings by the present author depicting the Ammon's horn sectors in accord with Lorente de Nó parceling (colored). (A)** Sagittal section through the dorsal hippocampus. Golgi-Cox technique. **(B)** Horizontal section through the ventral hippocampus of an adult rat. The actual width of the pyramidal cell layer as seen in Nissl-staining is shaded in gray. Golgi-Cox-Nissl technique. **(C)** Section obtained at a site comparable to that shown in “B” after the reduced-silver technique. Calibration bars = 200 μm.

**Figure 6:**
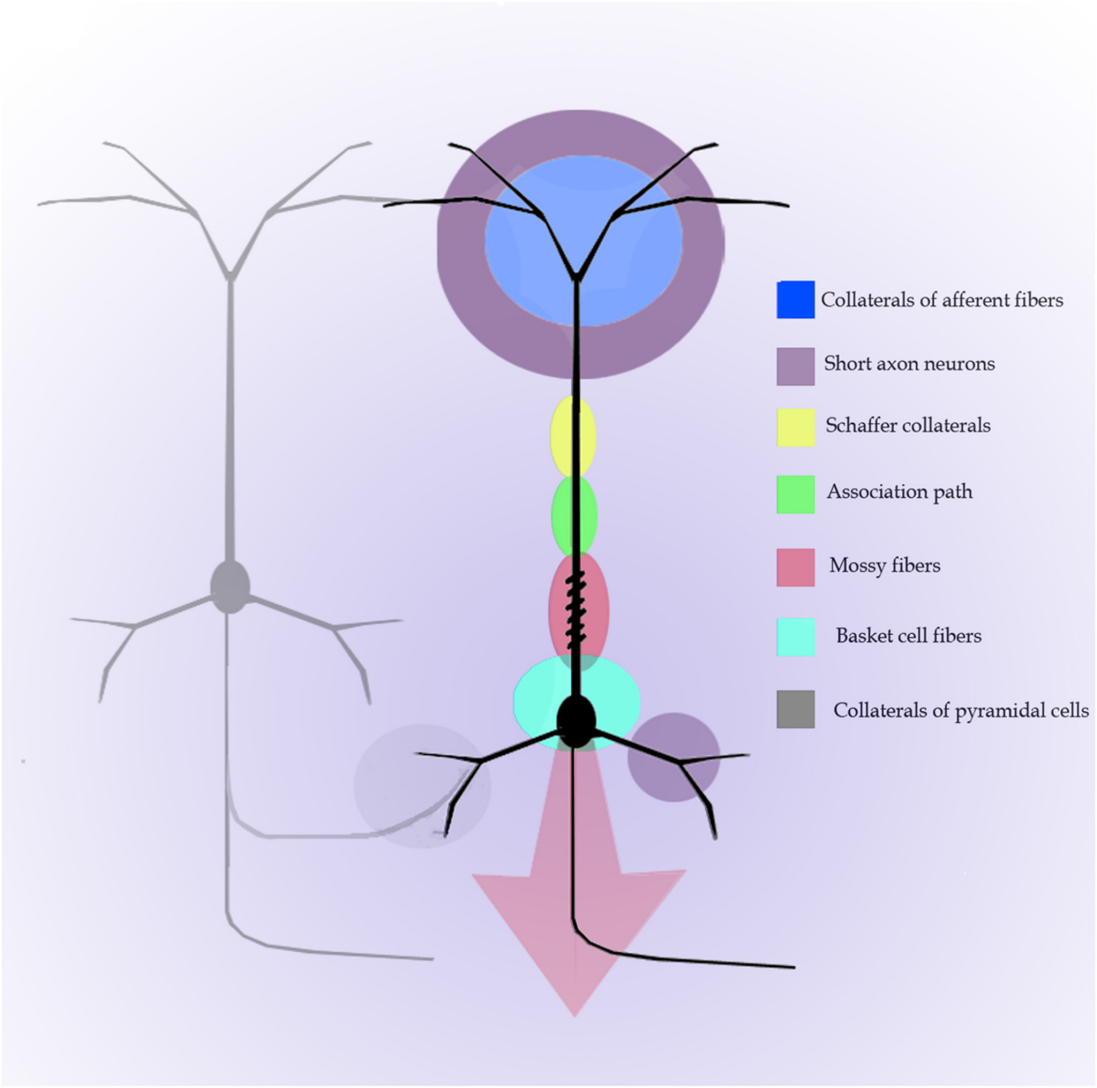
**Diagram of the distribution of afferences into distinct parts of the pyramidal cell of the Ammon's horn**. Modified and colored from Figure 34 in Lorente de Nó (1934).

In addition to the enduring contribution made by Lorente de Nó to the knowledge of inner structure and circuitry of the hippocampal formation (see Andersen et al., 2007), both the scholarly revision of previous work and the presentation of results he afforded, render this work an indispensable ingredient to both the novice and to the settled researcher. Possibly the best known contributions contained in this work are the plan of parceling of the Ammon's horn (AH) resulting in its current nomenclature (Figure 5) and the distribution of distinct afferences to the pyramidal cell (Figure 6). From Lorente de Nó's writing prior to this latter study it is clear that he was concerned with the proportions, location, and interactions of neurons. While Ramón y Cajal and Camilo Golgi performed their drawings from information kept in mind after observing the specimen, Lorente de Nó developed a variant of the Germanic approach. Researchers, particularly Oskar Vogt and his disciples, who were concerned with the proportions and dimensions of every layer or nuclei, utilized either camera lucida drawings or even photographic reproductions from large-format negatives. Lorente de Nó designed a hybrid system consisting of a projecting prism placed directly at the eye piece of the microscope. This straightforward procedure allow him to focus the microscopic image directly onto the working-table; then, the image was accurately copied (Figures 1–3); “every drawing should be a replica of a neuron,” he said (Larriva-Sahd, 2002). Following this method Lorente de Nó was able both to obtain a huge number of neuron samples and to detect subtle differences among pyramidal cells as a function of position from the entorhinal cortex to the dentate gyrus (Figures 5A,B). Moreover, he noted that, in addition to the well-known contribution of basket-cells to perisomatic axonal fibrils (Ramón y Cajal, 1904), afferences from each extrinsic [i.e., incoming tract(s)] (Figure 5C), and intrinsic (i.e., short-axon neurons) source distribute and terminate in different domains of the pyramidal cell (Figure 6). This cornerstone observation followed by his own interpretation shaped one of the fundamental concepts of current neurobiology. “… it has been possible to establish that the synapses of different kinds are not mixed but rather grouped in special regions of the cell; some on the body, some on the origin of the dendrites, some on the end, etc., although, of course the fields of distribution of the synapses of different kinds often overlap”(Figure 6). “But even without knowing what the multiplicity of connections really signify, one can conclude from the simple fact of its existence that the dendrites may not conduct in the same way as the axon, or in other words, that the central synapse cannot be compared with the neuro-muscular junction.” “The only possibility for cell Py1 (Figure 6) “using” all the impulses seems to be, first, that each synapse sets only a subliminal (chemical or other) change able to summation and second, that the conduction through the synapses is not follow by a refractory period. The subliminal changes are summated first in the dendrites and then in the surrounding of the axon takes place. When the change reaches the threshold value, an explosive discharge through the axon takes place axon. The axon–as well as any other nerve fiber–enters in a refractory state, but the cell body and dendrites do not do so, they continue receiving and adding subliminal changes until the threshold value is reached again and the axon has recovered.” In dimensioning implications of Lorente de Nó observations and tenets in this regard Swanson (1993) wrote: “This prediction is remarkably close to our modern view of central neuron physiology which awaited the impetus of the microelectrode (Brock et al., 1952). Nevertheless, as early as 1934, Lorente de Nó stated that the grater question in neurophysiology was how dendritic changes are summated, even though informed opinion at the time was skeptical of subthreshold summation in central neurons.” The recent implications of the distribution of afferents in the AH pyramidal cell are clearly presented by current authoritative authors (Somogyi and Klausberger, 2005), who wrote about the hippocampus: “These results suggest roles for specific interneuron types in structuring the activity of pyramidal cells via their respective target domains, and accurately timing and synchronizing pyramidal cell discharge, rather than providing generalized inhibition. Finally, interneurons belonging to different classes may fire preferentially at distinct time points during a given oscillation. As different interneurons innervate different domains of the pyramidal cells, the different domains will receive GABAergic input differentiated in time. Such a dynamic, spatio-temporal GABAergic control, which evolves distinct patterns during different brain states, is ideally suited to regulating the input integration of individual pyramidal cells contributing to the formation of cell assemblies and representations in the hippocampus and, probably, throughout the brain.”

## Concluding remark

It is obvious that Lorente de No's contributions, his observations, interpretations, and models of neurobiological events are widely utilized in contemporary neurobiology, often without citing his original works. To underscore their relevance, one could imagine what our understanding of neurobiology be without them. Granting that it is not reasonable to say that his explanations would not have been attained by anyone else, it would be difficult to imagine a coherent story about a neuron without sub-threshold excitability, a decoding nerve cell without temporo-spatial summation, or a cortical column without a neuronal substratum.

### Conflict of interest statement

The author declares that the research was conducted in the absence of any commercial or financial relationships that could be construed as a potential conflict of interest.
